# Grapevine Responses to the Entomopathogenic Fungi *Beauveria bassiana* and *Isaria fumosorosea* and the Effects of Salicylic Acid on Their Virulence Against the European Grapevine Moth, *Lobesia botrana*

**DOI:** 10.3390/microorganisms13071630

**Published:** 2025-07-10

**Authors:** Evangelos Beris, Xenophon Venios, Dimitrios Papachristos, Mathilde Ponchon, Dimitrios Kontodimas, Elias Korkas, Georgios Banilas, Annette Reineke

**Affiliations:** 1 Department of Wine, Vine and Beverage Sciences, School of Food Sciences, University of West Attica, 12243 Egaleo, Athens, Greece; 2 Laboratory of Agricultural Entomology, Department of Entomology and Agricultural Zoology, Benaki Phytopathological Institute, 14561 Kifissia, Athens, Greece; 3 Department of Crop Protection, Hochschule Geisenheim University, D-65366 Geisenheim, Germany

**Keywords:** *Vitis vinifera*, entomopathogenic fungi, *Beauveria bassiana*, *Isaria fumosorosea*, salicylic acid, physiological responses, spore germination, *Lobesia botrana*, larval mortality, mycosis

## Abstract

Entomopathogenic fungi (EPF) are substantial biocontrol agents reducing the populations of economically important pests in numerous crops. Recent findings indicate that their role in agroecosystems is more complex and extends to affecting plant physiology and growth. This study examined the effects of *Beauveria bassiana* and *Isaria fumosorosea*, as well as Salicylic acid (SA), on physiological parameters of grapevine (*Vitis vinifera* cv. Sauvignon Blanc). Additionally, the impact of SA on spore germination and pathogenicity of EPF against larvae of the European grapevine moth (*Lobesia botrana*) was tested. Foliar application of EPF was found to increase the electron transport rate (ETR) from PSII to PSI, indicating higher photosynthetic activity compared to control plants. EPF also elevated the transpiration rate (E) and stomatal conductance (gs). In contrast, SA treatments decreased E and gs, while the high dose (10 mM) exhibited reduced Fv/Fm value, accompanied by phytotoxic spots on leaves. Spore germination of both fungi was significantly reduced only by the SA concentration of 2 mM, while 0.5 and 1 mM did not affect germination. Combination EPF and SA treatments presented the highest larval mortality of *L. botrana* (87.5% at 28 °C and 77.5% at 24 °C for *B. bassiana* and *I. fumosorosea*, respectively). However, SA reduced larval mycosis in most cases. Overall, the results suggest that EPF and SA can be co-applied and included in vineyard integrated strategies to support grapevine health.

## 1. Introduction

Entomopathogenic fungi (EPF) are considered essential microbial biocontrol agents in sustainable agroecosystems, providing an ecologically friendly alternative to chemical pesticides [1,2]. Under favorable environmental conditions, EPF can effectively control populations of numerous economically important arthropod pests [3,4,5]. In ecological terms, EPF infect and eliminate insects by utilizing them as hosts to complete a phase of their life cycle [6]. Their infection process begins with conidial adhesion to the insect cuticle, followed by germination and enzymatic degradation of the exoskeleton via chitinases, proteases, and lipases, enabling hyphal penetration into the hemocoel [7,8].

Beyond pest control, EPF play a multifaceted role in agroecosystems, functioning as plant endophytes, rhizosphere colonizers, plant disease antagonists, and plant growth enhancers [9,10,11,12]. Lately, several methods of applying EPF as endophytes and the dual benefits of endophytic EPF (i.e., protecting tissues from pests and pathogens as well as promoting plant growth), have been reported in various plant species [13] including common bean [14], coffee [15], melon, and strawberry [16].

Yet, many aspects of the ecology and potential multifunctionality of EPF in vineyards remain unexplored. However, recent findings suggest that EPF may serve as beneficial microorganisms in vineyard environments, providing multiple advantages in complex ways. In a recent study, the entomopathogenic fungus *Metarhizium robertsii* (Ascomycota: Hypocreales) was applied to non-grafted vines (cv. Cabernet Sauvignon) and demonstrated significant protection against the grapevine phylloxera, *Daktulosphaira vitifoliae* (Hemiptera: Phylloxeridae) [17]. EPF has also been reported to colonize mature grapevines in a vineyard environment and persist as endophytes for at least five weeks, reducing infestation by piercing-sucking insects and leafhoppers [18]. Other recent studies highlight the importance of EPF in controlling key vineyard pests, such as the spotted wing drosophila, *Drosophila suzukii* (Diptera: Drosophilidae) [5,19], and the black vine weevil, *Otiorhynchus sulcatus* (Coleoptera: Curculionidae) [2,20]. Moreover, recent studies have demonstrated excellent performance of EPF as biocontrol agents of the European grapevine moth, *Lobesia botrana* (Lepidoptera: Tortricidae) [21,22,23,24,25,26]. Another study revealed that EPF can exhibit dual action by suppressing both *L. botrana* and the plant-pathogenic fungus *Eutypella microtheca* (Ascomycota: Xylariales) on grapevine [27]. Fungal entomopathogens have also been shown to exert additive and synergistic effects when combined with *Bacillus thuringiensis* for the control of *L. botrana* [28].

The European grapevine moth is currently the most severe pest in many viticultural regions across the globe [29,30,31]. Climate change is expanding its range into northern European wine regions, increasing its threat [32]. Beyond direct damage, *L. botrana* infestations promote secondary fungal infections such as *Botrytis*, further reducing grape yield and quality [33]. Control using chemical insecticides poses environmental and resistance risks [34], while also affecting indigenous yeasts and fermentation [35]. Alternative control strategies include mating disruption, egg parasitoids, and microbial biocontrol agents, with recent studies exploring nanotechnology and behavioral manipulation [36,37,38,39].

Salicylic acid (SA) is a naturally occurring phytohormone integral to various aspects of plant physiology, including growth regulation, stress mitigation, and disease resistance [40,41]. SA regulates the expression of genes involved in defense signaling pathways, thus contributing to enhanced plant immunity [42,43]. Except for mitigating various types of abiotic and biotic stress, exogenous application of SA has been reported to improve yield, growth, fruit quality, and postharvest capacity in many plant species [44,45,46]. SA also plays an important role in plant responses to herbivorous pests. Gene activation in response to herbivores depends on their feeding manner and tissue damage. Phloem-feeding whiteflies and aphids, causing minimal injury, trigger SA and Jasmonic acid (JA) ethylene-dependent signaling, similar to pathogen responses [47]. It has been recently found that SA positively regulates plant defenses against larvae of lepidopteran insects [48].

The main objectives of the present study were to investigate the physiological and phytotoxic effects of EPF and SA on grapevine plants, the potential influence of SA on spore germination of EPF, and their compatibility prospects for controlling larval populations of the European grapevine moth.

## 2. Materials and Methods

### 2.1. Biological Material (Plants, Fungal Isolates, Insects)

Three-year-old ‘Sauvignon Blanc’ vines (clone 108) were placed in a plant growth chamber (CMP-6050, Conviron, Winnipeg, MB, Canada) for budbreak and acclimation. The temperature was set at 25 °C and the relative humidity at 60–70%. The light intensity was ≈1000 μmol m^−2^ s^−1^, with a 13:11 photoperiod. Vines were grafted onto Richter 110 rootstock. They were growing in 8 L pots filled with a soil substrate mixture consisting of peat (70%), clay (20%), and zeolite (10%). Standard irrigation was applied every 48 h and moderate fertilization weekly (Nutri-Leaf 20-20-20, Miller Chemical & Fertilizer, Hanover, PA, USA).

The entomopathogenic fungal isolates *B. bassiana* IMI-391044 and *I. fumosorosea* EBAC-01 (Ascomycota: Hypocreales) were selected for their excellent performance in terms of spore germination, virulence, pest control, and compatibility with other agents, demonstrated in previous studies [28,49]. Fungi were sub-cultured in half-strength Sabouraud Dextrose Agar (SDA) for 20 days in the dark, upon initiation of the experiments.

Young third-instar larvae of *L. botrana* were reared as described in [26]. The semisynthetic diet that was used both for rearing and the bioassay consisted of: distilled water 1.4 L, corn flour 224 g, wheat germs 58 g, agar 32 g, brewing yeast 30 g, sorbic acid 8 g, Nipagin 4 g, Benzoic acid 4 g, formaldehyde 1.6 mL.

### 2.2. Chlorophyll a Fluorescence and Physiological Parameter Measurements

Spore suspensions of 1 × 10^7^ conidia/mL (in distilled water with 0.01% Tween 80) (RPI Corporation, Mt Prospect, IL, USA) were made for each fungus as described in [26]. Salicylic acid (Sigma-Aldrich, St. Louis, MO, USA) solutions (0.5, 2, and 10 mM) were prepared, also containing 0.01% Tween 80. The SA doses of 0.5 and 2 mM were selected as they have been previously studied in a range of fruit and vegetable crops, showing promising results [45,50]. All treatments were applied to plants as direct foliar sprays using 2 L. pressure sprayers (Master Ergo 2000, Marolex, Łomna, Poland). Control plants were sprayed with a standard 0.01% Tween 80 distilled water solution. Each treatment was replicated four times.

Measurements began 24 h post-spraying and were conducted twice a day, at light and dark periods. Chlorophyll a fluorescence measurements were performed using the LI-600 Fluorometer/Porometer (LI-COR Biosciences, Inc., Lincoln, NE, USA). Light-adapted state measurements were performed with the light intensity set at 1000 μmol m^−2^ s^−1^. The steady-state fluorescence (Fs) was determined, and thereafter, an 800 ms saturating light of 7500 µmol photons m^−2^ s^−1^ was applied to measure the maximum fluorescence in the light-adapted state (Fm′). The estimation of the actual quantum efficiency of PSII (ΦPSII) using the following formula:

(1) $$\Phi \mathrm{PSII}=\frac{\mathrm{Fm}\prime -\mathrm{Fs}}{\mathrm{Fm}\prime }$$

The electron transport rate (ETR) was calculated using the following equation:

(2) ETR= ΦPSII ∗ Qamb∗ abs∗PS2/1

where Qamb is ambient light, abs is the leaf light absorptance, and PS2/1 is the ratio of PSII to PSII absorptance. The parameters of maximum fluorescence (Fm) and minimum fluorescence (Fo) were determined, providing an 800 ms saturating pulse of continuous red light at 6000 µmol m^−2^ s^−1^, which was used for the calculation of the maximum quantum efficiency of PSII using the following equation:

(3) $$\frac{\mathrm{Fv}}{\mathrm{Fm}}=\frac{\mathrm{Fm}-\mathrm{Fo}}{\mathrm{Fm}}$$

Leaf temperature (Tleaf) measurements were conducted using a non-contact infrared thermometer (IRT) integrated into the LI-600 Porometer (LI-COR Biosciences, Inc., Lincoln, NE, USA). Transpiration rate (E) and stomatal conductance (gs) were computed from the difference in H_2_O in an air-stream flowing through a leaf cuvette using the following equations:

(4) $$Ε=\left(-\frac{\mathrm{Mr}\;(\mathrm{Ws}-\mathrm{Wr})}{S\;\left(1-\mathrm{Ws}\right)}\right)$$

where E is apparent transpiration (mol m^−2^ s^−1^), S is leaf area (m^2^), Mr (mol s^−1^) is the molar flow rate into the leaf cuvette, and Wr with Ws are water vapor mole fractions into and out of the leaf cuvette, respectively (mol H_2_O mol air^−1^).

(5) $$g_{\mathrm{sw}}=\frac{1}{\frac{1}{g_{\mathrm{tw}}}-\frac{1}{g_{\mathrm{blw}}}}$$

where g_sw_ is stomatal conductance (mol m^−2^ s^−1^), g_tw_ is total conductance to water vapor (mol m^−2^ s^−1^), and g_blw_ is boundary layer conductance (mol m^−2^ s^−1^).

Plants were daily checked for visible phytotoxicity for 14 days post-experiment. Photographs were captured using a digital camera (Canon Mirrorless EOS R50 18–45 mm, Canon Inc., Tokyo, Japan) and a stereomicroscope (SZX16, Olympus, Tokyo, Japan) at 14× Zoom with an adjusted EP50 camera.

### 2.3. Spore Germination Assays

Spore germination test of the entomopathogenic fungi *Beauveria bassiana* IMI-391044 and *Isaria fumosorosea* EBAC-01 was performed according to [49]. Briefly, fungi were cultured on half-strength SDA (CM0041, Oxoid™, Basingstoke, Hampshire, UK) for 20 days in the dark. Then, spores were harvested by scraping them using a microscope slide to produce suspensions of 1 × 10^5^ conidia/mL sterile distilled water with 0.01% Tween 80. Salicylic acid (Sigma-Aldrich) was mixed with half-strength SDA to produce agar plates with SA concentrations of 0.5, 1, and 2 mM. Therefore, SA treatments were integrated into the medium as suggested by [51,52]. 100 μL of the fungal liquid suspension was added to each agar plate and spread throughout the surface. Plates were incubated at 24 °C or 28 °C for 18 h (MLR-351H, Sanyo, Osaka, Japan). Each treatment consisted of four replicates (plates), and the entire experiment was repeated (*n* = 8). One group of 100 conidia was measured on each plate. Conidial germination was assessed by counting the percentage of germinated conidia (visible germ tubes). Measurements took place 12, 14, 16, and 18 h after the addition of the fungal suspension to the agar plates.

### 2.4. Larval Mortality Bioassay

This bioassay was conducted according to the methodology of [26]. Spore suspensions of 1 × 10^7^ conidia/mL water (sterile distilled water with 0.01% Tween 80) were made for each fungal isolate. Solutions of 0.5 and 1 mM SA (Sigma-Aldrich) in sterile distilled water with 0.01% Tween 80 were also prepared. Combination EPF and SA treatments were made by scraping and adding conidia to the respective SA solutions. A sterile distilled water solution of 0.01% Tween 80 was used for the control treatments. Third-instar larvae of *L. botrana* were treated by spraying them, using mini sprayers (BS-3: 3 Oz Personal Mini Sprayer, Sprayco^®^, Livonia, MI, USA) inside sterile Petri dishes with filter paper covering the bottom of each dish. After spraying, three cubes (approximately 1 cm^3^) of the semisynthetic diet were added to the plates. Then, they were covered with their lids, sealed with Parafilm^®^ (Bemis Inc., Neenah, WI, USA), and incubated either at 24 °C or 28 °C. Four replicates (consisting of ten larvae each) were utilized for each treatment, and the entire experiment was repeated.

Mortality measurements took place 3, 5, and 7 days after treatment. Any larva that was not moving, rotten, disintegrated, or presented symptoms of mycosis was recorded as dead. Immobility was confirmed by zero reaction to several contacts with sterile entomological pincers. After each mortality measurement, all dead insects were removed from the plates and transferred separately into new sterile Petri dishes containing a wet filter paper. After sealing with Parafilm^®^, the plates were kept at 25 °C for all treatments, and they were monitored daily for symptoms of mycosis. Mycosis was examined up to 14 days post-treatment according to the methodology of [53] to confirm fungal species.

### 2.5. Statistical Analysis

One-way analysis of variance (ANOVA) was performed to detect significant differences in all tested plant physiology parameters (maximum quantum efficiency and effective quantum efficiency of PSII, Stomatal conductance, ETR, transpiration rate, leaf temperature). One-way ANOVA was followed by Tukey’s HSD post hoc test to indicate significant differences between specific treatments. Two-way ANOVA was used to investigate whether the factors “treatment” and “temperature”, as well as the interaction between the two, presented significant effects on spore germination of EPF, larval mortality, and mycosis of *L. botrana*. Tukey’s HSD post hoc test was used to detect significant differences among treatments for each tested temperature regime. All statistical analyses were conducted using SPSS Statistics 29.

## 3. Results

### 3.1. Evaluation of Grapevine Physiological Responses upon Foliar Application of Entomopathogenic Fungi and Salicylic Acid

**Figure 1:**
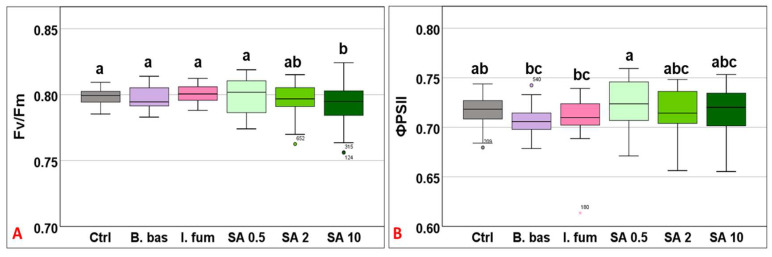
Box plot chart comparing maximum quantum efficiency (Fv/Fm) (**A**) and the effective quantum efficiency of PSII (ΦPSII) (**B**) among control plants (Ctrl) and plants treated with *B. bassiana* (*B. bas*) and *I. fumosorosea* (*I. fum*), as well as salicylic acid at 0.5 mM (SA 0.5), 2 mM (SA 2), and 10 mM (SA 10) solutions. Error bars represent mean  ±  standard deviation. Different lowercase letters denote statistically significant differences between treatments (*p* < 0.05).

Chlorophyll quenching analysis revealed that Fv/Fm mean values differed significantly across treatments (F_5,383_ = 5.18, *p* < 0.001). However, only the high concentration of SA (10 mM) differed significantly from the control (Figure 1A). ΦPSII mean values ranged from 0.707 to 0.722 (F_5,383_ = 4.68, *p* < 0.001), without significant differences among most treatments. However, *B. bassiana* and *I. fumosorosea* exhibited significantly lower values compared to SA 0.5 (*p* = 0.009) (Figure 1B).

**Figure 2:**
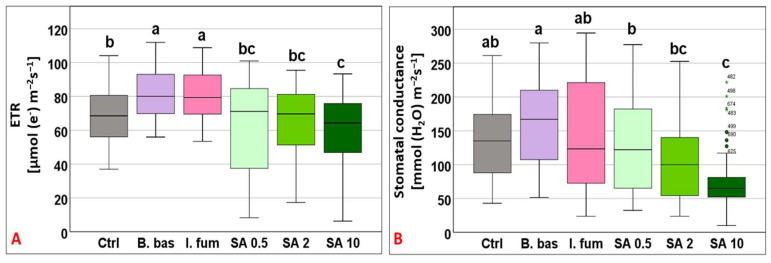
Box plot chart comparing electron transport rate (ETR) (**A**) and stomatal conductance (gs) (**B**) among control plants (Ctrl) and plants treated with *B. bassiana* (*B. bas*) and *I. fumosorosea* (*I. fum*), as well as salicylic acid at 0.5 mM (SA 0.5), 2 mM (SA 2), and 10 mM (SA 10) solutions. Error bars represent mean  ±  standard deviation. Different lowercase letters denote statistically significant differences between treatments (*p* < 0.05).

ETR varied greatly across treatments (F_5,383_ = 13.99, *p* < 0.001) and ranged from 60.1 to 82.08 μmol (e^−^) m^−2^ s^−1^. Plants sprayed with *B. bassiana* and *I. fumosorosea* exhibited the highest rates and differed significantly from the control (*p* < 0.001), whereas plants treated with SA 0.5, 2, and 10 mM presented the lower values (Figure 2A). The gs responses also displayed great fluctuations across treatments with mean values ranging from 75.36 to 162.81 mmol (H_2_O) m^−2^ s^−1^ (F_5,383_ = 15.77, *p* < 0.001), where *B. bassiana* and *I. fumosorosea* treated plants displayed the highest values and SA 10 mM the lowest mean value (*p* < 0.01) (Figure 2B).

**Figure 3:**
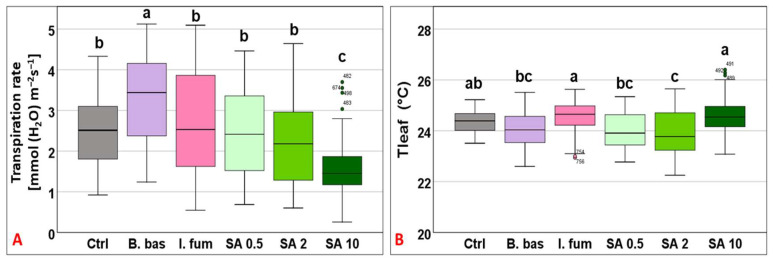
Box plot chart comparing transpiration rate (E) (**A**) and leaf temperature (Tleaf) (**B**) among control plants (Ctrl) and plants treated with *B. bassiana* (*B. bas*) and *I. fumosorosea* (*I. fum*), as well as salicylic acid at 0.5 mM (SA 0.5), 2 mM (SA 2), and 10 mM (SA 10) solutions. Error bars represent mean  ±  standard deviation. Different lowercase letters denote statistically significant differences between treatments (*p* < 0.05).

Transpiration followed a similar pattern to gs, ranging from 1.57 to 3.25 mmol (H_2_O) m^−2^ s^−1^ (F_5,383_ = 18.48, *p* < 0.001) with *B. bassiana* and *I. fumosorosea* exhibiting the highest performance, whereas SA 10 significantly had the lowest (*p* < 0.001) (Figure 3A). Tleaf values differed significantly across treatments (F_5,383_ = 10.9, *p* < 0.001) and ranged from 23.88 °C to 24.59 °C (Tgrowth = 25 °C). SA at 10 mM and *I. fumosorosea* treatments displayed the highest mean values, while significantly lower values (*p* < 0.001) were recorded for *B. bassiana*, SA 0.5, and SA 2 mM (Figure 3B).

**Figure 4:**
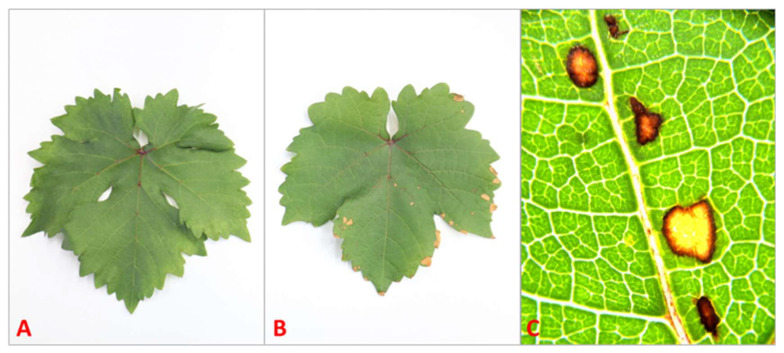
Leaves of Sauvignon Blanc: control (**A**), treated with 10 mM of salicylic acid (**B**), and necrotic spots caused by 10 mM of SA under stereomicroscope (SZX16 plus EP 50 digital camera, Olympus, Tokyo, Japan) at 14× magnification (**C**).

Macroscopic symptoms of phytotoxicity were only observed in plants treated with 10 mM SA. All plants of this category presented necrotic spots on sprayed leaves 48 h post-treatment (Figure 4).

### 3.2. Effects of SA on Spore Germination of EPF

**Figure 5:**
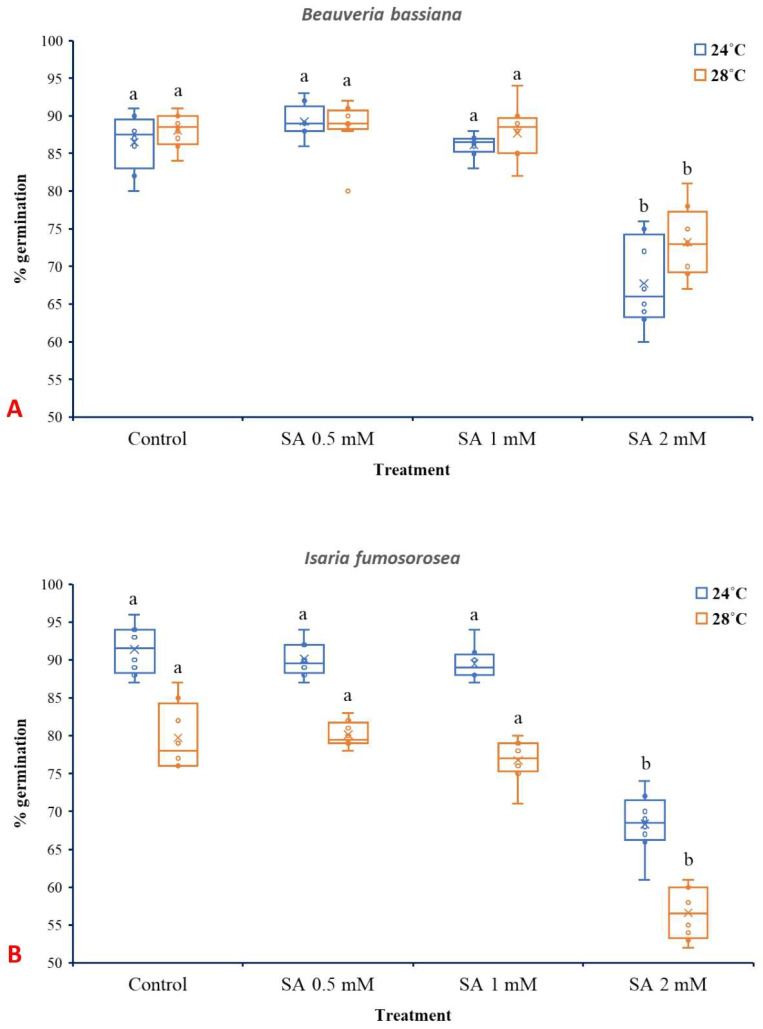
Box plots presenting the median values with upper and lower quartiles. The whiskers represent the range of the variabilities outside the quartiles, and the outliers are plotted as individual points. The × represents the mean percentage of spore germination of *B. bassiana* IMI-391044 (**A**) and *I. fumosorosea* EBAC-01 (**B**) in half-strength SDA agar (control), fortified with 0.5, 1, and 2 mM of SA, after 18 h of incubation at 24 °C or 28 °C. Different letters indicate significant differences (*p* < 0.05) for each temperature.

SA at 2 mM significantly reduced spore germination of *B. bassiana* and *I. fumosorosea*, while lower concentrations (0.5 and 1 mM) did not present significant effects. In the case of *B. bassiana* (Figure 5A), temperature (24 °C or 28 °C) and treatment (SA 0.5, 1, and 2 mM) individually presented significant influence (*p* ˂ 0.001), while their interaction did not show a significant effect (F_3,56_ = 1.929, *p* = 0.135). Similar to *B. bassiana,* both the different temperatures and SA treatments had significant effects in the case of *I. fumosorosea* (*p* ˂ 0.001) (Figure 5B), while their interaction was not significant (F_3,56_ = 0.514, *p* = 0.674).

### 3.3. Effects of SA on Larval Mortality of L. botrana Caused by EPF

**Figure 6:**
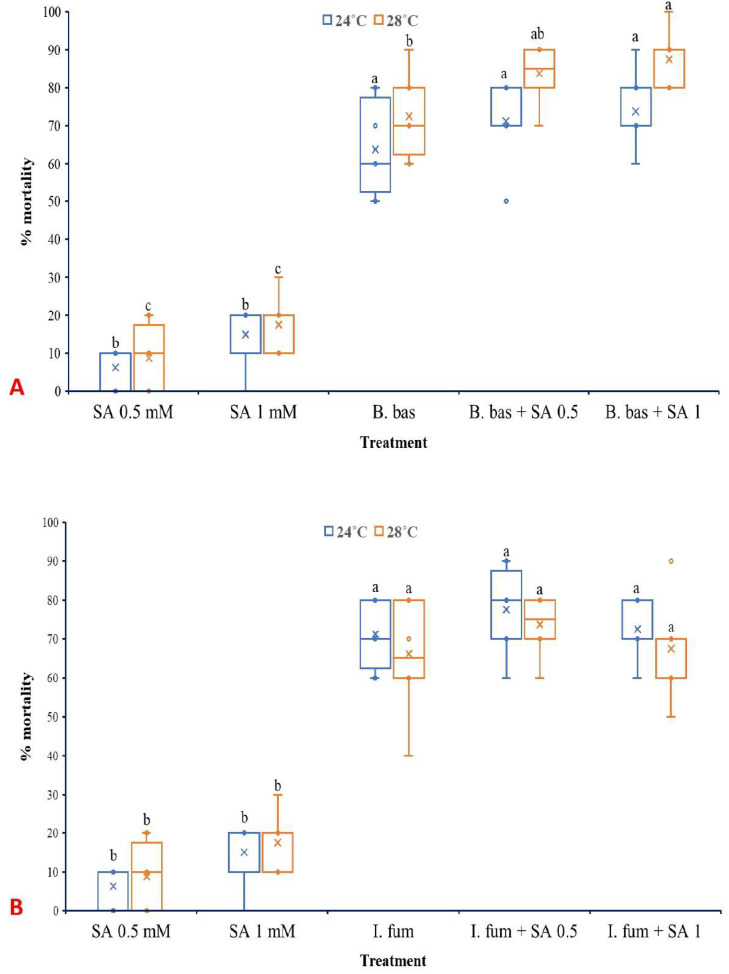
Box plots presenting the median values with upper and lower quartiles. The whiskers represent the range of the variabilities outside the quartiles, and the outliers are plotted as individual points. The × represents the mean percentage of *L. botrana* larval mortality caused by *B. bassiana* IMI-391044 (**A**) and *I. fumosorosea* EBAC-01 (**B**), in single or combination treatments with SA, seven days post-treatment. Different letters indicate significant differences (*p* < 0.05) for each temperature.

Both temperature (F_1,70_ =17.314, *p* < 0.001) and treatment (F_4,70_ = 268.798, *p* < 0.001) had significant effects on the mortality of *L. botrana* larvae caused by *B. bassiana* (Figure 6A). However, the interaction between them did not present a significant impact (F_4,70_ =1.547, *p* = 0.198). *B. bassiana* caused high levels of mortality to *L. botrana* larvae at 28 °C (72.5%) and even higher when combined with exogenous SA at 0.5 mM (83.75%) and 1 mM (87.5%).

In the case of *I. fumosorosea*, only treatment influenced significantly the rates of larval mortality (F_4,70_ =211.083, *p* < 0.001), while temperature (F_1,70_ =0.747, *p* = 0.390) as well as the interaction between the factors (F_4,70_ =0.747, *p* = 0.563) did not exhibit significant effects (Figure 6B). Control treatments presented zero mortality of *L. botrana* larvae at both temperature regimes, while single SA treatments caused low levels of larval mortality.

**Figure 7:**
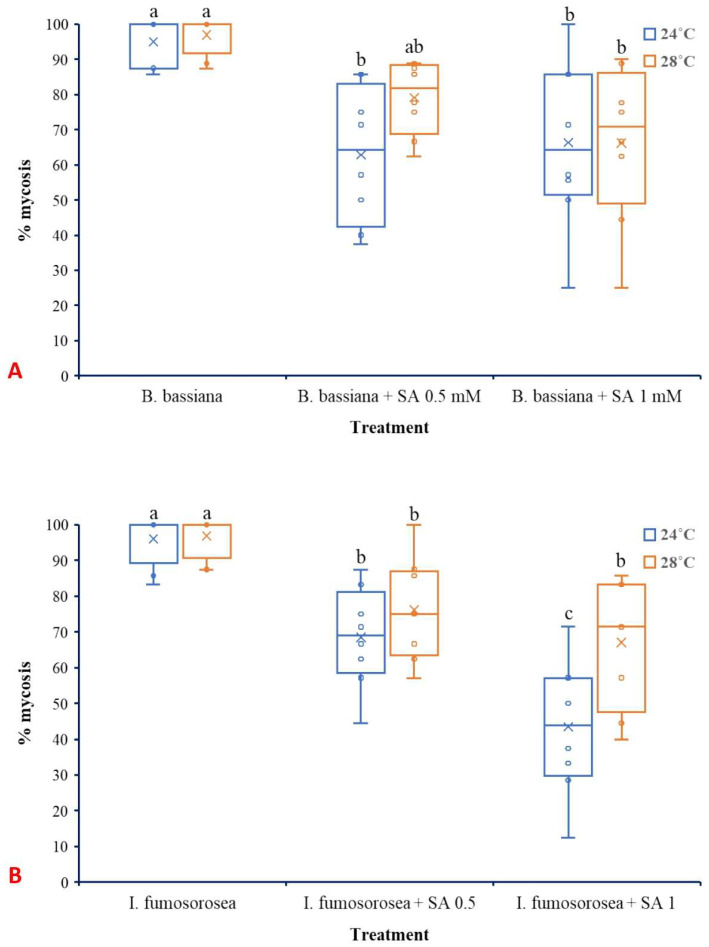
Box plots presenting the median values with upper and lower quartiles. The whiskers represent the range of the variabilities outside the quartiles, and the outliers are plotted as individual points. The × represents the mean percentage of *L. botrana* larval mycosis caused by *B. bassiana* IMI-391044 (**A**) and *I. fumosorosea* EBAC-01 (**B**) 14 days post-treatment. Different letters indicate significant differences (*p* < 0.05) for each temperature.

Regarding mycosis that was developed on dead *L. botrana* larvae, temperature did not significantly affect either *B. bassiana* (F_1,42_ =1.621, *p* = 0.210) (Figure 7A) or *I. fumosorosea* (F_1,42_ =0.747, *p* = 0.390) (Figure 7B). Oppositely, treatment significantly influenced mycosis caused by both *B. bassiana* (F_2,42_ =15.003, *p* ˂ 0.001) and *I. fumosorosea* (F_2,42_ =211.083, *p* ˂ 0.001). The interaction between factors did not present significant effects for *B. bassiana* (F_2,42_ =1.163, *p* = 0.323) as well as for *I. fumosorosea* (F_2,42_ =0.747, *p* = 0.563).

## 4. Discussion

Chlorophyll quenching analysis revealed no signs of photoinhibition or phytotoxic stress effects in most treatments, with Fv/Fm values remaining close to the optimal range (0.8), suggesting healthy photosynthetic apparatus without serious suppression [54]. The only exception was the high concentration of SA (10 mM), which caused a reduction in Fv/Fm values accompanied by notable phytotoxic spots on leaves.

Leaves sprayed with either *B. bassiana* or *I. fumosorosea* spores exhibited increased electron transport rate (ETR) and a slight downregulation of the quantum efficiency of photosystem II (ΦPSII). This may reflect an adaptive strategy to reallocate photochemical energy and enhance ATP synthesis, possibly through the activation of cyclic electron flow (CEF), thereby supplying additional energy for the activation of defense mechanisms [55]. The observed gs increase further supports this hypothesis, indicating increased energy requirements for metabolic activity that promote CO_2_ uptake and stimulate photosynthetic activity [56], as indicated by increased ETR. All these findings support a metabolic reprogramming scenario toward the activation of defense mechanisms induced by endophytic colonization, without the presence of photochemical stress, as evidenced by the high Fv/Fm values. In other studies, the presence of EPF has been reported to promote growth in several plant species under certain conditions [57,58,59,60].

In contrast, SA-treated plants exhibited significant stomatal closure responses, accompanied by subsequent decreases in photosynthetic activity, which was more prominent at high SA concentrations. In a previous study conducted using a different grapevine cultivar, a low concentration of SA (0.1 mM) did not significantly influence the net photosynthesis rate at moderate temperatures but alleviated declines in Photosynthesis and Rubisco activation state caused by heat stress [61]. In our study, although SA induced an anti-transpiratory effect, leaf temperature (Tleaf) was lower in SA-treated plants compared to those sprayed with EPF, implying a better leaf thermoregulation. With the exception of the high SA dose (10 mM), where the reduction in transpiration was dramatic, the plants treated with low (0.5 mM) or medium (2 mM) SA concentrations exhibited the lowest Tleaf, which may not be explained solely by their low transpiration rates. This suggests an improved thermoregulatory leaf capacity, with reduced water loss, which may be attributed to diminished metabolic heat production, as well as potentially more effective leaf cooling mechanisms [62].

Results demonstrated that SA at 2 mM significantly inhibits the spore germination of both *B. bassiana* and *I. fumosorosea*, indicating its potent antifungal activity at rather high concentrations. Similar inhibitory effects of SA on fungal germination and growth have been documented previously [63,64,65]. Interestingly, lower concentrations of SA (0.5 or 1 mM) did not significantly affect spore germination, while in some cases, 0.5 mM SA slightly enhanced germination in *B. bassiana*. This observation may reflect a hormetic response, where low doses of a stressor stimulate biological activity, a phenomenon reported in various fungal systems [66].

Temperature also significantly influenced spore germination in both fungal species, aligning with previous results on the thermal sensitivity of entomopathogenic fungi [49,67,68]. The absence of a significant interaction between temperature and SA treatment shown here suggests that the SA’s inhibitory effect is consistent across the temperature range tested. Notably, *I. fumosorosea* exhibited greater sensitivity to SA than *B. bassiana*, which may be attributed to species-specific differences. Spore germination of *I. fumosorosea* at 24 °C ranged from 91% (control) to 68% (SA 2 mM). These findings are in accordance with the results of [51], where spore germination of *I fumosorosea* was also inhibited by SA, although different media and SA concentrations were tested.

Both *EPF* at spore concentrations of 1 × 10^7^ conidia/mL caused exceptionally high levels of larval mortality at both 24 °C and 28 °C, demonstrating strong potential as biocontrol agents of *L. botrana*. These results corroborate previous studies in which EPF demonstrated strong efficacy against *L. botrana* [23,24,26]. Humidity is well known to play a substantial role in germination and virulence of EPF in pest control [69,70]. The relative humidity in this study was kept at a moderate and consistent level across treatments to minimize its influence and allow the effects of other factors to emerge more distinctly. The present study showed that the application of SA, particularly at rather low concentrations, does not inhibit the larvicidal action of EPF against *L. botrana*. In contrast, low SA concentrations were shown even to enhance the efficacy of *B. bassiana* against *L. botrana*. Mortality levels in the combined EPF and SA treatments were generally higher than those observed in single EPF treatments, indicating a possible synergistic interaction. Previous findings have shown that SA can modulate insect physiology in ways that increase vulnerability to microbial pathogens, potentially by interfering with cuticular defenses or immune function [71].

Interestingly, although mortality increased, the incidence of mycosis was reduced in the combined treatments compared to single EPF applications. This inverse relationship suggests that SA, while facilitating early infection processes or weakening host resistance, may subsequently hinder the ability of the fungus to colonize the host cadaver and sporulate. In this study, SA presented significant inhibition of late-stage fungal growth. However, further studies are needed to confirm this action using histological or molecular methods. Nevertheless, when SA is applied at appropriate doses and under optimum pH values is well-documented to possess antifungal properties, and such effects have been shown to suppress post-infection development of fungi [63,72]. The differential response between *B. bassiana* and *I. fumosorosea*, with the former being more responsive to SA treatment in terms of increased mortality, may be attributed to species-specific virulence strategies and compatibility with plant-derived compounds. Similar species-level differences have been reported in fungal responses to plant signaling molecules and environmental stressors [7].

## 5. Conclusions

Findings highlight the complex interplay between EPF and salicylic acid in grapevine physiology and pest control. While high concentrations of SA induced phytotoxicity and inhibited fungal germination, lower doses demonstrated compatibility with EPF applications and even enhanced their insecticidal efficacy against *L. botrana* in some cases. The absence of photoinhibition and the stimulation of photosynthetic activity in EPF-treated plants suggest a beneficial endophytic interaction, possibly linked to induced resistance mechanisms. Notably, the observed increase in larval mortality followed by reduced mycosis in combination treatments indicates a dual function of SA: enhancing host susceptibility to EPF infection while subsequently restricting fungal colonization and sporulation. These results emphasize the potential of combining low doses of SA with EPF in integrated pest management strategies. However, the observed variation in fungal responses and the dose-dependent effects of SA highlight the need for precise optimization to achieve consistent and effective outcomes. Further research is needed to elucidate the interactions between EPF and SA within pest management strategies and to assess their potential benefits for grapevine performance under abiotic stress conditions.

## Data Availability

The original contributions presented in this study are included in the article. Further inquiries can be directed to the corresponding authors.

## Abbreviations

The following abbreviations are used in this manuscript:

EPF	Entomopathogenic fungi
SA	Salicylic acid
ETR	Electron transport rate
PSII	Photosystem II
PSI	Photosystem I
E	Transpiration rate
gs	Stomatal conductance
Fv/Fm	Maximum quantum efficiency of PSII
IPM	Integrated pest management
JA	Jasmonic acid
Fs	steady-state fluorescence
Fm′	maximum fluorescence in the light-adapted state
ΦPSII	actual quantum efficiency of PSII
Qamb	Ambient light
abs	Leaf light absorptance
PS2/1	Ratio of PSII to PSII absorptance
Fm	maximum fluorescence
Fo	minimum fluorescence
Tleaf	Leaf temperature
IRT	non-contact infrared thermometer
S	leaf area
Mr	molar flow rate into the leaf cuvette
Wr	water vapor mole fractions into the leaf cuvette
Ws	water vapor mole fractions out of the leaf cuvette
gtw	total conductance to water vapor
gblw	boundary layer conductance
CEF	cyclic electron flow

## References

[1] Roy H.E., Brodie E.L., Chandler D., Goettel M.S., Pell J.K., Wajnberg E., Vega F.E. (2010). Deep Space and Hidden Depths: Understanding the Evolution and Ecology of Fungal Entomopathogens. BioControl.

[2] Khun K.K., Wilson B.A.L., Stevens M.M., Huwer R.K., Ash G.J. (2020). Integration of Entomopathogenic Fungi into IPM Programs: Studies Involving Weevils (Coleoptera: Curculionoidea) Affecting Horticultural Crops. Insects.

[3] Lee W.W., Shin T.Y., Bae S.M., Woo S.D. (2015). Screening and Evaluation of Entomopathogenic Fungi against the Green Peach Aphid, *Myzus persicae*, Using Multiple Tools. J. Asia Pac. Entomol..

[4] Qasim M., Su J., Noman A., Ma T., Islam W., Hussain D., Rizwan M., Hameed M.S., Khan K.A., Ghramh H.A. (2024). Citrus Psyllid Management by Collective Involvement of Plant Resistance, Natural Enemies and Entomopathogenic Fungi. Microb. Pathog..

[5] Quesada-Moraga E., González-Mas N., Yousef-Yousef M., Garrido-Jurado I., Fernández-Bravo M. (2024). Key Role of Environmental Competence in Successful Use of Entomopathogenic Fungi in Microbial Pest Control. J. Pest Sci..

[6] Vivekanandhan P., Kannan S., Pittarate S., Krutmuang P. (2024). Classification, Biology and Entomopathogenic Fungi-Based Management and Their Mode of Action against *Drosophila* Species (Diptera: Drosophilidae): A Review. Front. Microbiol..

[7] Zimmermann G. (2007). Review on Safety of the Entomopathogenic Fungi *Beauveria bassiana* and *Beauveria brongniartii*. Biocontrol Sci. Technol..

[8] Shahid A., Rao Q., Bakhsh A., Husnain T. (2012). Entomopathogenic Fungi as Biological Controllers: New Insights into Their Virulence and Pathogenicity. Arch. Biol. Sci..

[9] Vega F.E., Goettel M.S., Blackwell M., Chandler D., Jackson M.A., Keller S., Koike M., Maniania N.K., Monzón A., Ownley B.H. (2009). Fungal Entomopathogens: New Insights on Their Ecology. Fung. Ecol..

[10] Lacey L.A., Grzywacz D., Shapiro-Ilan D.I., Frutos R., Brownbridge M., Goettel M.S. (2015). Insect Pathogens as Biological Control Agents: Back to the Future. J. Invertebr. Pathol..

[11] Kumar P., Joshi A., Sharma N., Lata S., Mehmood S., Ahlawat Y.K., Malik A., Moussa I.M., Kerketta A., Soni P. (2024). Integrative Approaches to Improve Litchi (*Litchi chinensis* Sonn.) Plant Health Using Bio-Transformations and Entomopathogenic Fungi. BMC Plant Biol..

[12] Vivekanandhan P., Alford L., Krutmuang P. (2024). Editorial: Role of Entomopathogenic Fungi in Sustainable Agriculture. Front. Microbiol..

[13] Bamisile B.S., Siddiqui J.A., Akutse K.S., Ramos Aguila L.C., Xu Y. (2021). General Limitations to Endophytic Entomopathogenic Fungi Use as Plant Growth Promoters, Pests and Pathogens Biocontrol Agents. Plants.

[14] Parsa S., Ortiz V., Vega F.E. (2013). Establishing Fungal Entomopathogens as Endophytes: Towards Endophytic Biological Control. J. Vis. Exp..

[15] Martins J.L.A., Franzin M.L., Ferreira D.d.S., Magina L.C.R., Martins E.F., Mendonça L.V.P., Neves W.d.S., Pallini A., Valicente F.H., Schmidt J.M. (2024). *Metarhizium*-Inoculated Coffee Seeds Promote Plant Growth and Biocontrol of Coffee Leaf Miner. Microorganisms.

[16] Mantzoukas S., Daskalaki E., Kitsiou F., Papantzikos V., Servis D., Bitivanos S., Patakioutas G., Eliopoulos P.A. (2022). Dual Action of *Beauveria bassiana* (Hypocreales; Cordycipitaceae) Endophytic Stains as Biocontrol Agents against Sucking Pests and Plant Growth Biostimulants on Melon and Strawberry Field Plants. Microorganisms.

[17] Ponchon M., Reineke A., Massot M., Bidochka M.J., Thiéry D., Papura D. (2022). Three Methods Assessing the Association of the Endophytic Entomopathogenic Fungus *Metarhizium robertsii* with Non-Grafted Grapevine *Vitis vinifera*. Microorganisms.

[18] Rondot Y., Reineke A. (2018). Endophytic *Beauveria bassiana* in Grapevine *Vitis vinifera* (L.) Reduces Infestation with Piercing-Sucking Insects. Biol. Control.

[19] Galland C.D., Lalaymia I., Declerck S., Verheggen F. (2023). Efficacy of Entomopathogenic Fungi against the Fruit Fly *Drosophila Suzukii* and Their Side Effects on Predator (*Orius laevigatus*) and Pollinator (*Bombus terrestris*) Insects. Entomol. Gen..

[20] Pope T.W., Hough G., Arbona C., Roberts H., Bennison J., Buxton J., Prince G., Chandler D. (2018). Investigating the Potential of an Autodissemination System for Managing Populations of Vine Weevil, *Otiorhynchus sulcatus* (Coleoptera: Curculionidae) with Entomopathogenic Fungi. J. Invertebr. Pathol..

[21] Aguilera Sammaritano J., Deymié M., Herrera M., Vazquez F., Cuthbertson A.G.S., López-Lastra C., Lechner B. (2018). The Entomopathogenic Fungus, *Metarhizium anisopliae* for the European Grapevine Moth, *Lobesia botrana* Den. & Schiff. (Lepidoptera: Tortricidae) and Its Effect to the Phytopathogenic Fungus, *Botrytis Cinerea*. Egypt J. Biol. Pest Control.

[22] Altimira F., De La Barra N., Rebufel P., Soto S., Soto R., Estay P., Vitta N., Tapia E. (2019). Potential Biological Control of the Pupal Stage of the European Grapevine Moth *Lobesia Botrana* by the Entomopathogenic Fungus *Beauveria pseudobassiana* in the Winter Season in Chile. BMC Res. Notes.

[23] López Plantey R., Papura D., Couture C., Thiéry D., Pizzuolo P.H., Bertoldi M.V., Lucero G.S. (2019). Characterization of Entomopathogenic Fungi from Vineyards in Argentina with Potential as Biological Control Agents against the European Grapevine Moth *Lobesia botrana*. BioControl.

[24] Altimira F., De La Barra N., Godoy P., Roa J., Godoy S., Vitta N., Tapia E. (2021). *Lobesia botrana*: A Biological Control Approach with a Biopesticide Based on Entomopathogenic Fungi in the Winter Season in Chile. Insects.

[25] Arias-Aravena M., Altimira F., Gutiérrez D., Ling J., Tapia E. (2022). Identification of Exoenzymes Secreted by Entomopathogenic Fungus *Beauveria pseudobassiana* RGM 2184 and Their Effect on the Degradation of Cocoons and Pupae of Quarantine Pest *Lobesia botrana*. JoF.

[26] Beris E., Papachristos D., Ponchon M., Caca D., Kontodimas D., Reineke A. (2024). The Effects of Temperature on Pathogenicity of Entomopathogenic Fungi for Controlling Larval Populations of the European Grapevine Moth (*Lobesia botrana*) (Lepidoptera: Tortricidae). Crop Prot..

[27] Aguilera-Sammaritano J., Caballero J., Deymié M., Rosa M., Vazquez F., Pappano D., Lechner B., González-Teuber M. (2021). Dual Effects of Entomopathogenic Fungi on Control of the Pest *Lobesia botrana* and the Pathogenic Fungus *Eutypella microtheca* on Grapevine. Biol. Res..

[28] Beris E., Korkas E. (2021). Additive and Synergistic Interactions of Entomopathogenic Fungi with *Bacillus thuringiensis* for the Control of the European Grapevine Moth *Lobesia botrana* (Denis and Schiffermüller) (Lepidoptera: Tortricidae). Egypt J. Biol. Pest Control.

[29] Andreadis S.S., Milonas P.G., Savopoulou-Soultani M. (2005). Cold Hardiness of Diapausing and Non-diapausing Pupae of the European Grapevine Moth, *Lobesia botrana*. Entomol. Exp. Appl..

[30] Moreau J., Villemant C., Benrey B., Thiéry D. (2010). Species Diversity of Larval Parasitoids of the European Grapevine Moth (*Lobesia botrana*, Lepidoptera: Tortricidae): The Influence of Region and Cultivar. Biol. Control.

[31] Benelli G., Lucchi A., Anfora G., Bagnoli B., Botton M., Campos-Herrera R., Carlos C., Daugherty M.P., Gemeno C., Harari A.R. (2023). European Grapevine Moth, *Lobesia botrana* Part I: Biology and ecology. Entomol. Gen..

[32] Iltis C., Moreau J., Pecharová K., Thiéry D., Louâpre P. (2020). Reproductive Performance of the European Grapevine Moth *Lobesia botrana* (Tortricidae) Is Adversely Affected by Warming Scenario. J. Pest Sci..

[33] Pasquini S., Haxaire-Lutun M.O., Rison J.-L., Flier W.G., Teixeira L.A. (2018). Susceptibility of *Lobesia botrana* (Lepidoptera: Tortricidae) to Chlorantraniliprole in the Emilia Romagna Region of Northeast Italy. J. Econ. Entomol..

[34] Civolani S., Boselli M., Butturini A., Chicca M., Fano E.A., Cassanelli S. (2014). Assessment of Insecticide Resistance of *Lobesia botrana* (Lepidoptera: Tortricidae) in Emilia-Romagna Region. J. Econ. Entomol..

[35] Caboni P., Cabras P. (2010). Pesticides’ Influence on Wine Fermentation. Advances in Food and Nutrition Research.

[36] Ioriatti C., Anfora G., Tasin M., De Cristofaro A., Witzgall P., Lucchi A. (2011). Chemical Ecology and Management of *Lobesia botrana* (Lepidoptera: Tortricidae). J. Econ. Entom..

[37] Lucchi A., Sambado P., Juan Royo A.B., Bagnoli B., Conte G., Benelli G. (2018). Disrupting Mating of *Lobesia botrana* Using Sex Pheromone Aerosol Devices. Environ. Sci. Pollut. Res..

[38] Benelli G., Lucchi A., Anfora G., Bagnoli B., Botton M., Campos-Herrera R., Carlos C., Daugherty M.P., Gemeno C., Harari A.R. (2023). European Grapevine Moth, *Lobesia botrana* Part II: Prevention and Management. Entomol. Gen..

[39] Benelli G., Pavoni L., Zeni V., Ricciardi R., Cosci F., Cacopardo G., Gendusa S., Spinozzi E., Petrelli R., Cappellacci L. (2020). Developing a Highly Stable *Carlina acaulis* Essential Oil Nanoemulsion for Managing *Lobesia botrana*. Nanomaterials.

[40] Koo Y.M., Heo A.Y., Choi H.W. (2020). Salicylic Acid as a Safe Plant Protector and Growth Regulator. Plant Pathol. J..

[41] Liu J., Qiu G., Liu C., Li H., Chen X., Fu Q., Lin Y., Guo B. (2022). Salicylic Acid, a Multifaceted Hormone, Combats Abiotic Stresses in Plants. Life.

[42] Ali S., Ganai B.A., Kamili A.N., Bhat A.A., Mir Z.A., Bhat J.A., Tyagi A., Islam S.T., Mushtaq M., Yadav P. (2018). Pathogenesis-Related Proteins and Peptides as Promising Tools for Engineering Plants with Multiple Stress Tolerance. Microbiol. Res..

[43] Song W., Shao H., Zheng A., Zhao L., Xu Y. (2023). Advances in Roles of Salicylic Acid in Plant Tolerance Responses to Biotic and Abiotic Stresses. Plants.

[44] Rivas-San Vicente M., Plasencia J. (2011). Salicylic Acid beyond Defence: Its Role in Plant Growth and Development. J. Exp. Bot..

[45] Dobón-Suárez A., Giménez M.J., García-Pastor M.E., Zapata P.J. (2021). Salicylic Acid Foliar Application Increases Crop Yield and Quality Parameters of Green Pepper Fruit during Postharvest Storage. Agronomy.

[46] Chen C., Sun C., Wang Y., Gong H., Zhang A., Yang Y., Guo F., Cui K., Fan X., Li X. (2023). The Preharvest and Postharvest Application of Salicylic Acid and Its Derivatives on Storage of Fruit and Vegetables: A Review. Sci. Hortic..

[47] Walling L.L. (2000). The Myriad Plant Responses to Herbivores. J. Plant Growth Regul..

[48] Setotaw Y.B., Li J., Qi J., Ma C., Zhang M., Huang C., Wang L., Wu J. (2024). Salicylic Acid Positively Regulates Maize Defenses against Lepidopteran Insects. Plant Divers..

[49] Beris E. (2021). Evaluation and Environmental Testing of Entomopathogenic Fungi for Their Effectiveness as Bio-Control Agents of Major Vineyard Pests. Plant Prot..

[50] Giménez M.J., Valverde J.M., Valero D., Guillén D., Martínez-Romero D., Serrano M., Castillo S. (2014). Quality and Antioxidant Properties on Sweet Cherries as Affected by Preharvest Salicylic and Acetylsalicylic Acids Treatments. Food Chem..

[51] Vega F.E., Dowd P.F., McGuire M.R., Jackson M.A., Nelsen T.C. (1997). In VitroEffects of Secondary Plant Compounds on Germination of Blastospores of the Entomopathogenic Fungus *Paecilomyces fumosoroseus* (Deuteromycotina: Hyphomycetes). J. Invertebr. Pathol..

[52] Strobel N.E., Porter L.A. (2005). Salicylate Inhibits Growth of Plant-Pathogenic Fungi and Synergistically Enhances the Activity of Other Antifungal Materials In Vitro. J. Ky. Acad. Sci..

[53] Onsongo S.K., Mohamed S.A., Akutse K.S., Gichimu B.M., Dubois T. (2022). The Entomopathogenic Fungi *Metarhizium anisopliae* and *Beauveria bassiana* for Management of the Melon Fly *Zeugodacus cucurbitae*: Pathogenicity, Horizontal Transmission, and Compatability with Cuelure. Insects.

[54] Sánchez-Moreiras A.M., Graña E., Reigosa M.J., Araniti F. (2020). Imaging of Chlorophyll a Fluorescence in Natural Compound-Induced Stress Detection. Front. Plant Sci..

[55] Moustaka J., Meyling N.V., Hauser T.P. (2021). Root-Associated Entomopathogenic Fungi Modulate Their Host Plant’s Photosystem II Photochemistry and Response to Herbivorous Insects. Molecules.

[56] Oue H. (2023). Comparisons of the Stomatal Conductance and Electron Transport Rate of Three Japanese Rice Cultivars Including Himenorin in Ehime Prefecture. J. Agric. Meteorol..

[57] Ahsan S.M., Injamum-Ul-Hoque M., Das A.K., Rahman M.M., Mollah M.M.I., Paul N.C., Choi H.W. (2024). Plant–Entomopathogenic Fungi Interaction: Recent Progress and Future Prospects on Endophytism-Mediated Growth Promotion and Biocontrol. Plants.

[58] Wilberts L., Rojas-Preciado N., Jacquemyn H., Lievens B. (2023). Fungal Strain and Crop Cultivar Affect Growth of Sweet Pepper Plants after Root Inoculation with Entomopathogenic Fungi. Front. Plant Sci..

[59] Liu Y., Yang Y., Wang B. (2022). Entomopathogenic Fungi *Beauveria bassiana* and *Metarhizium anisopliae* Play Roles of Maize (*Zea mays*) Growth Promoter. Sci. Rep..

[60] Chaudhary P.J., B.L. R., Patel H.K., Mehta P.V., Patel N.B., Sonth B., Dave A., Bagul S.Y., M. D., Jain D. (2023). Plant Growth-Promoting Potential of Entomopathogenic Fungus *Metarhizium pinghaense* AAUBC-M26 under Elevated Salt Stress in Tomato. Agronomy.

[61] Wang L.-J., Fan L., Loescher W., Duan W., Liu G.-J., Cheng J.-S., Luo H.-B., Li S.-H. (2010). Salicylic Acid Alleviates Decreases in Photosynthesis under Heat Stress and Accelerates Recovery in Grapevine Leaves. BMC Plant Biol..

[62] Gonçalves F.C.D.M., Mantoan L.P.B., Corrêa C.V., Parreiras N.D.S., De Almeida L.F.R., Ono E.O., Rodrigues J.D., Prado R.D.M., Boaro C.S.F. (2024). Effects of Salicylic Acid on Physiological Responses of Pepper Plants Pre-Subjected to Drought under Rehydration Conditions. Plants.

[63] Da Rocha Neto A.C., Maraschin M., Di Piero R.M. (2015). Antifungal Activity of Salicylic Acid against *Penicillium expansum* and Its Possible Mechanisms of Action. Int. J. Food Microbiol..

[64] Li L., Zhu T., Song Y., Feng L., Kear P.J., Riseh R.S., Sitohy M., Datla R., Ren M. (2022). Salicylic Acid Fights against *Fusarium* Wilt by Inhibiting Target of Rapamycin Signaling Pathway in *Fusarium oxysporum*. J. Adv. Res..

[65] Gacnik S., Munda A., Veberic R., Hudina M., Mikulic-Petkovsek M. (2023). Preventive and Curative Effects of Salicylic and Methyl Salicylic Acid Having Antifungal Potential against *Monilinia laxa* and the Development of Phenolic Response in Apple Peel. Plants.

[66] Calabrese E.J., Baldwin L.A. (2003). Hormesis: The Dose-Response Revolution. Annu. Rev. Pharmacol. Toxicol..

[67] Mishra S., Kumar P., Malik A. (2015). Effect of Temperature and Humidity on Pathogenicity of Native *Beauveria bassiana* Isolate against *Musca domestica* L. *J*. Parasit. Dis..

[68] Athanassiou C.G., Kavallieratos N.G., Rumbos C.I., Kontodimas D.C. (2017). Influence of Temperature and Relative Humidity on the Insecticidal Efficacy of *Metarhizium anisopliae* against Larvae of *Ephestia kuehniella* (Lepidoptera: Pyralidae) on Wheat. J. Insect Sci..

[69] Lazzarini G.M.J., Rocha L.F.N., Luz C. (2006). Impact of Moisture on in Vitro Germination of *Metarhizium anisopliae* and *Beauveria bassiana* and Their Activity on *Triatoma infestans*. Mycol. Res..

[70] Bohatá A., Folorunso E.A., Lencová J., Osborne L.S., Mraz J. (2024). Control of Sweet Potato Whitefly (*Bemisia tabaci*) Using Entomopathogenic Fungi under Optimal and Suboptimal Relative Humidity Conditions. Pest Manag. Sci..

[71] Mollah M.M.I., Choi H.W., Yeam I., Lee J.M., Kim Y. (2021). Salicylic Acid, a Plant Hormone, Suppresses Phytophagous Insect Immune Response by Interrupting HMG-Like DSP1. Front. Physiol..

[72] Yang J., Wang Y., Liu L., Liu L., Wang C., Wang C., Li C. (2019). Effects of Exogenous Salicylic Acid and pH on Pathogenicity of Biotrophy-Associated Secreted Protein 1 (BAS1)-Overexpressing Strain, *Magnaporthe oryzae*. Environ. Sci. Pollut. Res..

